# Curcumin mitigates the sleep-deprivation impacts on rat hypothalamic paraventricular nucleus

**DOI:** 10.1016/j.ibneur.2023.11.008

**Published:** 2023-11-29

**Authors:** Mahboobeh Erfanizadeh, Ali Noorafshan, Mohammad Reza Namavar, Saied Karbalay-Doust, Tahereh Talaei-Khozani

**Affiliations:** a Department of Anatomical Sciences, School of Medicine, Shiraz University of Medical Sciences, Shiraz, Iran; b Histomorphometry and Stereology Research Center, Shiraz University of Medical Sciences, Shiraz, Iran; c Clinical Neurology Research Center, Shiraz University of Medical Sciences, Shiraz, Iran; d Laboratory for stem cell research, Department of Anatomical Sciences, Shiraz University of Medical Sciences, Shiraz, Iran

**Keywords:** Sleep deprivation, Paraventricular nucleus, Stereology, Spatial arraignment, Apoptosis, Curcumin

## Abstract

**Introduction:**

The paraventricular nucleus of the hypothalamus (PVH) is an important efferent system that relays the circadian rhythm of sleep and stress information to the periphery. Chronic REM sleep deprivation (CSD) is thought to damage this system. We evaluated the effects of CSD after 21 days on the spatial arrangement of PVH in male rats and the anti-apoptotic effects of curcumin on cell loss in sleep-deprived rats.

**Methods:**

The rats received 1 mL of 100 mg/kg/day of curcumin in 3 groups: the CSD (through a modified multiple platform apparatus, 18 h/day), grid-floor control, and cage-control along with the same set of matched groups which received 1 mL PBS. In the grid-floor control group, as a control for CSD, animals were placed on stainless-steel-mesh grids positioned upon the CSD apparatus and then allowed to sustain the chance to sleep. After 21 days, their brains were removed for stereological estimations, Voronoi tessellation, and TUNEL assay. In an unbiased stereological approach, Cavalieri’s principle and an optical disector were used for estimating the volume and total cell number of the PVH, respectively. The Voronoi tessellation was measured using Image J software.

**Results:**

Significant reductions (*P* < 0.05) in the PVH volume and cell number, along with an increase in dead neurons, were found in CSD animals. The spatial pattern of two types of PVH neurons (parvocellular and magnocellular) showed random distributions after CSD, whereas curcumin not only increased the volume and neuronal number but also retrieved the spatial distribution to a regular one.

**Conclusions:**

CSD decreased the volume and altered the spatial arrangement of the neurons in PVH by increasing apoptosis and decreasing the cell number. However, oral use of curcumin could protect PVH from these changes.

## Highlights

• Curcumin co-treatment could not prevent weight loss induced by CSD.
• CSD increases the apoptotic neurons and curcumin co-treatment decreases it.
• CSD reduces the PVH volume and cell number but curcumin co-treatment prevents it.
• Randomly distributed PVH neurons in the CSD change to regular by curcumin co-treatment.

## 1 Introduction

In humans, sleep loss can have widespread detrimental effects on health and is a risk factor for several neurodegenerative diseases including Alzheimer's disease, related dementia, and Parkinson's disease (Leng et al., 2019). PVH, located in the ventral diencephalon (Machluf et al., 2011) immediately behind the anterior commissure, regulates the stress response (Füzesi et al., 2016), learning, and memory (Li et al., 2017). Chronic stress results in hyper-reactivity of the hypothalamic-pituitary-adrenocortical axis, weight loss, and anhedonia (Miklós and Kovács, 2012). The glutamatergic neurons in the paraventricular nucleus of the hypothalamus (PVH) play a unique role in sleep-wake regulation (Liu et al., 2020). The PVH relays information from the suprachiasmatic nucleus to the autonomic system via the superior cervical ganglion (Teclemariam-Mesbah et al., 1997). The postganglionic fibers project to the pineal gland via Conary nerves to regulate the nocturnal secretion of melatonin (Amaral and Cipolla-Neto, 2018). This hormone is mostly used to regulate the circadian rhythms and the sleep/wake cycle. All the activities controlled by PVH are adversely affected by acute and chronic sleep deprivation (Aguirre, 2016, Fifel et al., 2018).

Chronic REM sleep deprivation (CSD, sleep deprivation during 21 days) is related to deficits in attention, cognition, mood, immune function, and metabolism (During and Kawai, 2017, Moreno-Villanueva et al., 2018). The effects of CSD have been shown on the morphology, volume, cell number, and viability in various parts of the nervous system including the brain cortex (Noorafshan et al., 2017), its nuclei (Kamali et al., 2017), and sensory and autonomic ganglia (Erfanizadeh et al., 2020, Verkhratsky et al., 2019).

Acute sleep deprivation can intensely impair both decision-making functions and cognitive learning (Lim and Dinges, 2010) and alter the metabolic hormonal balance (Zhu et al., 2019).

Previous studies have indicated that REM sleep has a protective role for neurons from damage and apoptosis (Biswas et al., 2006), then it is reasonable that REM sleep deprivation may cause cell death. Neuronal apoptosis has been seen following CSD (Noorafshan et al., 2017). To model the lifestyle of night shift workers, a CSD protocol was used in this study, in which REM sleep deprivation was induced. Many neurodegenerative diseases are associated with loss of rapid eye movement sleep (REMS). REMS loss increases noradrenaline (NA) levels and damages mitochondria causing the release of cytochrome c to activate the intrinsic pathway to induce neuronal apoptosis and degeneration (Somarajan et al., 2016).

Apoptosis occurs by CSD, as one of the major pathways in cell death (Cao et al., 2019), and DNA fragmentation (Ranjan et al., 2010). Additionally, an increase of NLRP3 inflammasome (an intracellular sensor that detects endogenous danger signals, and environmental irritants) after CSD mediated the pyroptosis of the neurocyte which is a highly inflammatory mode of regulated cell death (Fan et al., 2021).

Anti-apoptotic components, including botanicals, may alleviate the adverse effects of CSD. Curcumin (an active component of turmeric) is an FDA-approved component with antioxidant and anti-apoptotic effects (Noorafshan et al., 2017), which crosses the blood-brain barrier and is neuroprotective in neurological disorders. A previous study has shown that curcumin inhibits cellular damage and apoptosis by diminishing the endoplasmic reticulum stress (Zhou et al., 2022) and lessening malondialdehyde levels and anti-apoptotic mechanisms (Zhao et al., 2010). So it may prevent potentially harmful structural changes in the PVH induced by CSD.

With regard to the above considerations, we examined, for the first time, the possible destructive effects of CSD and the protective impact of curcumin on the morphology, volume and cell number, cell survival as well as the spatial pattern of neuronal distance.

## 2 Materials and methods

### 2.1 Animals and drugs

A total of 36 Sprague-Dawley (250–300 g) male adult rats were used for this study. The current study stemmed from the same group of animals used in our previous paper (Erfanizadeh et al., 2020), which investigated the effect of curcumin and CSD on the superior sympathetic ganglion. All procedures were done based on the guidelines on standard animal care and approved by the local ethical committee (IR.SUMS.REC.1396. S630). All animals were housed under standard conditions (22–24 °C and 12:12 h light-dark cycles), with ad libitum access to food and water. Animals were divided randomly into six groups including cage-Control, Grid floor-control, CSD, curcumin, grid floor + curcumin, and CSD+ curcumin. The cage-control and Curcumin groups were placed in the glass cages. The CSD and CSD+ curcumin rats were placed in the water tank with modified multiple platforms to induce CSD. As suitable control for the CSD-induced groups, the grid floor and grid floor + curcumin animals were kept in a water tank with a stainless-steel grid mesh put on the round platforms (Kamali et al., 2017). 100 mg/kg/day of curcumin (CN 8203540010, Sigma-Aldrich) in PBS (Zhang et al., 2022) was gavaged to all the curcumin-treated groups. The other groups were gavaged with the same volume of PBS (1 mL/day). The experiments were performed over a 21-day period.

### 2.2 Induction of CSD

CSD groups were placed in separate water tanks and paradoxical sleep deprivation was done for them using the modified multiple platform method (MMPM). CSD was induced by placing six rats in a plexiglas water tank (125 ×45 ×45 cm) containing 14 round small platforms (6.5 cm in diameter). The MMPM deprives the animals from sleep by falling into water due to muscle relaxation in rapid eye movement (REM) sleep; thus, non-REM sleep declines in this method (da Silva Rocha-Lopes et al., 2018, Machado et al., 2013). The MMPM was designed to reduce individual stress; a recovery time window was included by providing social communication among animals without hindering free movement (Kamali et al., 2017). The CSD was inflicted for 21 days, through which animals were placed daily in the tank for 18 h (16:00–10:00). On the next day, the rats were returned to their cages and allowed to sleep a 6-hour window (10:00–16:00). In MMPM, the tank was filled with water about 1 cm below the surface of the platforms or the grid at ambient temperature before 16:00. The lights-on plan for the CSD boxes was in line with the light/dark cycle, similar to the control and grid-floor control animals. The animals in the grid-floor control group were placed on the top of the round platforms, and the surface of the tank was covered by a stainless-steel meshwork as a grid floor. The grid floor prevented their falling into the water-induced stress without sleep deprivation and could be considered as the control for the CSD group (Noorafshan et al., 2018). For habituation, each animal in the CSD and grid-floor control groups was placed in the apparatus and subjected to sleep restriction protocol for a period of 30 min daily for 5 days, prior to the CSD experiment.

### 2.3 The stereological methods

Animals were sacrificed by deep anesthesia. The whole brain was fixed by buffer formalin, the right and left cerebral hemispheres were cut on the mid-sagittal plane, and the paraffin-embedded blocks were prepared. The right cerebral hemisphere was serially sectioned at 26 µm, and we left one at 5 µm thicknesses.

### 2.4 Estimation of the volume

PVH was identified in Giemsa-stained 26-µm thick sections according to the rat brain atlas (Paxinos and Watson, 2006). Every four or five sections containing PVH was selected for stereological quantitation (Bregma ∼1.8–1.88 mm posterior to Bregma). The volume of the PVH was estimated using point-counting based on Cavalieri’s principle. Systematic random sampling was applied to the sample in fixed intervals determined in advance (8–12 sections in each nucleus). The live image of each section was assessed at a final magnification of 110× using the stereological software (Stereo Lite, SUMS, Shiraz, Iran). The volume of each PVH (V _(nucleus)_) was estimated by the following formula:

V(nucleus)=∑A(sections)×dWhere ∑ A is the sum area of the sections and d is the interval between the sampled sections (Uylings et al., 2005).

#### 2.4.1 Estimation of the neuron and glial cell number

**Fig. 1:**
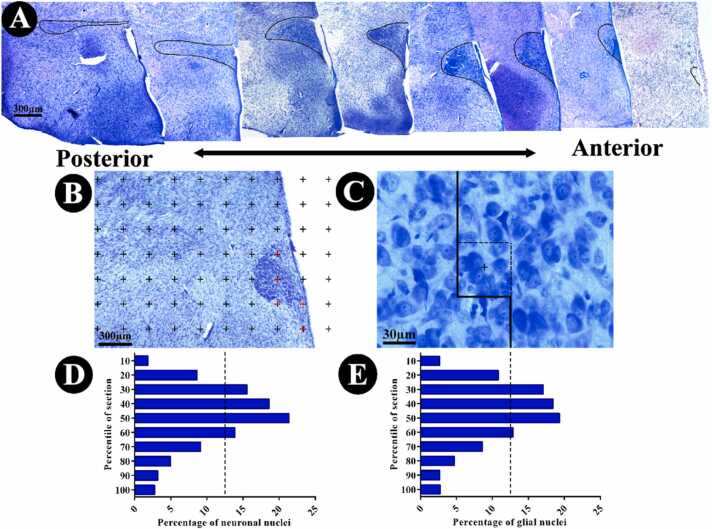
Stereological methods for evaluating the quantitative features at the paraventricular nucleus of the hypothalamus (PVH). (A) Position of PVH in the ventral diencephalon adjacent to the third ventricle (B) For the Cavalieri estimate of total PVH volume, the superimposed point-counting grid and all test points hitting the section were counted. (C) An optical disector snapshot. Neurons whose nuclei came into focus during the scanning of the disector’s height without touching the left and bottom borders of the frame were counted (arrow). (D) The Z-axis distribution of the neuronal nuclei and (E) glial nuclei was plotted to define the height of the disector. There are 10 columns, each representing the percentage of the counted nuclei in 10% of the section thickness from the top to bottom sections.

The number of neurons and glial cells of the right PVH was counted in the sections whose volume was calculated, using the optical disector method. We used the basic morphology of a normal neuron in Nissl staining consisting of a large cell body or perikaryon with the neurites (dendrites and axon) emerging from the cell body, Nissl substance in perikaryon (and dendrites), and the identifiable nucleus that is invariably pale or euchromatic with discrete nucleolus (Namavar et al., 2020). Also, a narrow rim of the cytoplasm circling the entire nucleus represents a valuable feature to distinguish the small neurons from the astrocytes (García-Cabezas et al., 2016). The endothelial cells y were identified by a rectangular nucleus with rounded corners, shaping of the nuclei to the tubular shape of capillaries, cytoplasm without staining, and granules in the nuclear envelope and heterochromatin network. Other cells that were not identified as neurons or endothelial cells were considered to be glial cells with small and dark nuclei without nucleoli and stained cytoplasm (Bjugn and Gundersen, 1993, García-Cabezas et al., 2016). A computer linked to a light microscope (Nikon E200, Nikon, Japan) with an oil immersion lens (60×, numerical aperture: 1.4) was employed to estimate the total number of neurons and glial cells. According to the “optical disector” technique, the microscopic fields were scanned and sampled at equal distances in X and Y directions to ensure systematic uniform random sampling (Deniz et al., 2018). The movement of the microscope stage on the Z-axis was measured using a microcator (MT12, Heidenhain, Traunreut, Germany) (Kristiansen and Nyengaard, 2012). The unbiased counting frames with an area (“*a/f*”) of 1016 µm^2^ were used for both cell types. To earn the suitable guard area and the height of the disector (h), we plotted the Z-axis distribution from the nuclei. Briefly, the counted nuclei were grouped in 10 columns through the height of the tissue (calculated by the microcator) from the top (0%) downward (100%) (Fig. 1). The upper 10% and lower 20% of the cell nuclei were ignored based on the histogram and regarded as the guard zones. Height is obtained in each column based on the mean focus distance to un-focus in all counted fields. Therefore, cell counting was carried out at the remaining 78% of cell nuclei within (*h*) and corrected for overestimation (Pakkenberg and Gundersen, 1988). Any nuclei entering the focus within the sampling box (h multiplied by a/f) were selected if it was located totally or even partly inside the counting frame and also did not touch the unacceptable lines (left and bottom borders of the frame) (Fig. 1). The total number of the neuron and the glial cell was estimated by means of multiplying the numerical density (N*v*) and V (PVH):

$$\mathrm{Nv}\left(\mathrm{cells}⁄\mathrm{unit}\;\mathrm{volume}\;\right)=\left[\frac{\sum Q^{-}}{\sum P\times \left(a/f\right)\times h}\times \frac{t}{\mathrm{BA}}\right]$$

Where “ΣQ^−^” was the total number of the nuclei coming into focus throughout scanning the height of the disector (Fig. 1); “ΣP”, the total number of counting frames in all counted fields; “h”(14 µm), the height of the disector; “a/f”, the frame area; “t”, the mean section thickness calculated in every sampled field using the microcator (23 µm within the average); and “BA”, the block advance of the microtome set at 26 µm (Deniz et al., 2018).

#### 2.4.2 Estimation of the coefficient of error

The coefficient of error (CE) for the volume and number estimation was calculated based on Gunderson et al.’s method (1999) (Howard and Reed, 2004). The coefficient of error (CE) for the estimated volume “CE (V)” is the function of the noise effect and systematic random sampling variance for the sum of areas. Since the cross-sectional area “ΣA” was estimated by the software, CE (V) was calculated through the following formula:

$$CE\left(V\right)={\left(\sum A\right)}^{-1}\times [1/12\times {\left(3\sum AiAi+\sum AiAi+2-4\sum AiAi+1\right)}^{1/2}$$

The precision of the optical disector in estimating the cell number per animal is expressed by the coefficient of error (CE). The CE for the estimated total neuron number, CE (N), was derived from CE (V) and CE (Nv), using the following formula:

$$CE(N\nu )={\left[\left(\frac{n}{n-1}\right)\times \left[\frac{{\left(\sum Q^{-}\right)}^{2}}{\sum Q^{-}\sum Q^{-}}+\frac{{\left(\sum P\right)}^{2}}{\sum P\sum P}-\frac{2\sum \left(Q^{-}P\right)}{\sum Q^{-}\sum P}\right]\right]}^{\frac{1}{2}}$$

$$CE\left(N\right)=\left[\sqrt{CE^{2}(N\nu )+CE^{2}\left(V\right)}\right]$$

### 2.5 TUNEL assay

5-μm-thick sections on superfrost slides were subjected to TUNEL assay, using the In Situ Cell Death Detection Kit, POD (Cat. No 11684817910, Roche Diagnostics, Indianapolis, USA) according to the manufacturer’s protocol. Briefly, tissue sections were deparaffinized, and endogenous peroxidase was blocked by treatment with methanol containing 3% H_2_O_2_ for 10 min. The slides were rinsed in PBS, and DNA decondensation was performed by incubating them in a 50 µg/mL Proteinase K (Roche Diagnostics, Indianapolis, USA) in10mM Tris-HCl solution, pH 7.4–8.0 for 45 min at 37 °C. The sections were incubated with the TUNEL reaction mixture for 60 min at 37 °C. TUNEL reaction was stopped by rinsing the sections in distilled water. The sections were observed by fluorescent (Olympus BX51) microscopes (Olympus DP73, Japan) at 450–500 nm. Then, converter-POD was added, and the samples were incubated at 37 °C for 30 min. The sections were then incubated with 0.5 mg/mL diaminobenzidine (Sigma-Aldrich, D5905) in 0.05 mol/L TBS (pH 7.6) containing 0.02% H_2_O_2_ for 10 min and counterstained with 0.1% fast green. Negative controls were performed using a labeled solution instead of a TUNEL reaction mixture. The rat thymus with Hydrocortisone-induced apoptosis thymocyte was used as the positive control (Odaka and Mizuochi, 2002). For semi-quantitative analysis, the percentage of TUNEL-positive neurons was recorded in each group (n = 6, and 10 fields for each sample). The apoptotic index was determined by the following equation (Mutiah et al., 2016):

$\mathrm{Apoptotic Index}=\frac{\mathrm{apoptotic cell}}{\mathrm{total cell}}\times 100\%$

### 2.6 Evaluation of spatial distribution of the neurons

According to Giemsa staining, PVH neurons were divided into two general categories of magnocellular (large polyhedral cells filled with Nissl granules) and parvocellular (small rounded cells) based on the site and size of soma (Lu et al., 2001). Voronoi tessellation has great utility with many applications (Quey and Renversade, 2018) including geometric modeling biology (Bock et al., 2010). The spatial distribution of parvocellular and magnocellular neurons in the PVH was evaluated separately using Voronoi tessellation.

Voronoi tessellation is a decomposition of 2D planes into independent polygons that correspond to the area occupied by a single cell, and the size of the area depends on the distance between the neighboring cells. Therefore, a statistical analysis of the polygonal areas provides morphometric information about the spatial distribution pattern of the cells. The area and number of closest Voronoi polygons to each other were then obtained. To draw the Voronoi polygon diagram, we analyzed the tissue sections (with a 40 × objective lens) of nuclei using the video-microscopy system. The images were analyzed using the Voronoi Plugin in Image J (Java. NIH, USA) (van Horssen et al., 2013). The coefficient of variation or CV (standard deviation of the polygon areas/ mean×100) provides an indication of the spatial distribution of the neurons: CV of 33–64% is associated with a random distribution, less than 33% has a regular distribution, and more than 64% is considered as a clustered distribution (Duyckaerts and Godefroy, 2000, Moroni et al., 2008). This CV is a classification and not a statistical comparison(Sotoudeh et al., 2020).

### 2.7 Statistical analysis

Data are presented as Mean ± SEM. The Kolmogorov-Smirnov test was done to normalize the data. To measure the body weight, analysis of the volumes, glial cell numbers, and apoptotic cells percentile, we performed a one-way ANOVA assay, followed by appropriate Tukey’s post-hoc test for parametric (normality and equal variance passed) data. Kruskal-Wallis test based on the ranks was used followed by Dunn’s post-hoc test for nonparametric (normality and/or equal variance failed) data. The neuronal numbers were analyzed using a 2-tailed Mann-Whitney-U test. For the area of Voronoi polygons, the Brown-Forsythe test was used to determine the equality of variances, and a one-way ANOVA was conducted to assess the overall statistical significance differences. *P* values <0.05 were considered as statistically significant. GraphPad Prism 6 Demo (Version 6.07, © 1995–2015 GraphPad Software, Inc., USA) was used for all analyses.

## 3 Results

### 3.1 The histomorphological assay

**Fig. 2:**
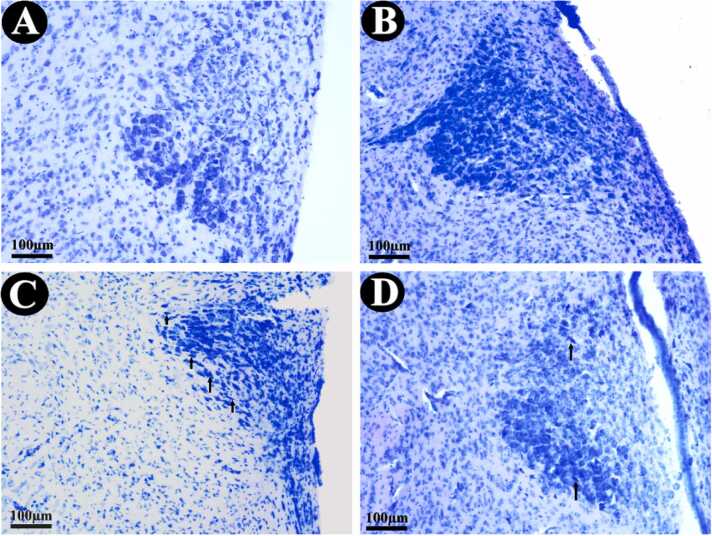
Representative photograph of the PVH. A normal appearance can be seen in the cage-control group (A). Higher neuron number in curcumin-treated animals (B). A smaller population and shrinking of the neurons as well as increase of pyknotic cells (arrows) can be found in the CSD animals (C). Normal histology appeared in the PVH of the animals exposed to both CSD and curcumin. The arrow indicates pyknotic cell (D). The other groups were almost similar to the A and B.

The histological appearance of the control, grid, and grid/curcumin-treated groups was statistically similar. The number of neurons was higher in the curcumin than in the control group. In addition, the CSD animals showed a lower cell population and pyknotic cells compared to the control group. Curcumin administration seems to have recovered the pathological changes in CSD-exposed animals, but pyknotic cells are still seen (Fig. 2). Pyknotic cells were identified by their highly stained nuclei with peripheral chromatin condensations (C-shaped) or one or more solid darkly stained bodies (Amrein et al., 2004).

### 3.2 The volume of the PVH

**Fig. 3:**
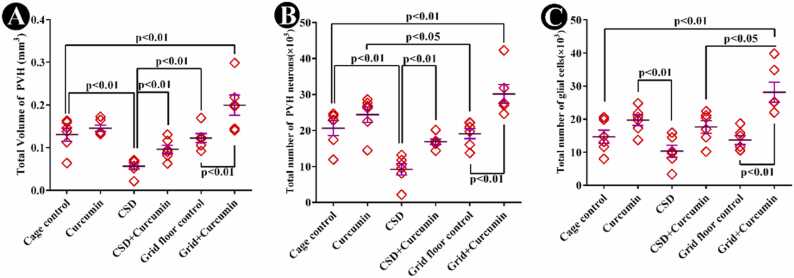
Dot plots showing stereological findings in the PVH. (A) The total volume of PVH in different groups. CSD significantly decreased the PVH volume, and it improved significantly with curcumin (*P* < 0.01). (B) The total number of neurons of PVH. The CSD led to a significant decrease in this parameter, and it was retrieved significantly by treating with curcumin (*P* < 0.01). (C) The total number of the glial cells of PVH decreased in the CSD in comparison with the control group non-significantly and treatment with curcumin significantly improved it (*P* < 0.01). The lines over each dot plot represent mean ± SEM (standard error of the mean).

Both cage-control and grid-floor groups showed a statistically similar volume. The volume of the PVH was reduced in the CSD group when compared to the control group (*P* < 0.01). However, the CSD group that received curcumin, showed an increase in the volume in comparison with the CSD group (*P* < 0.01), and the volume of PVH in the CSD + curcumin group was similar to the control animals. The grid-floor-treated animals that received curcumin also showed an increase in the PVH volume in comparison with the grid-floor control group (*P* < 0.01). The range of CE of the estimated volume was 0.04–0.05 for all groups (Fig. 3A).

### 3.3 The number of neurons

In the PVH, the total neuronal number was almost similar in both grid-floor and cage-control groups. The neuronal number was reduced in the CSD group compared to the control group (*P* = 0.0043). The number of neurons increased in the CSD animals treated with curcumin when compared to the CSD group (*P* = 0.0022) to a degree that was similar to the control group, which indicated its protective effect. Also, the number of neurons in the grid floor animals that received curcumin was higher than those in the cage-control group (*P* = 0.0043) and grid floor control group (*P* = 0.0022). The neuronal number increased in the curcumin group compared to the grid floor control group (*P* = 0.04). The range of CE of the estimated number of neurons was 4–6% for all groups (Fig. 3B).

### 3.4 The number of glial cells

The total number of glial cells in both grid-floor and cage-control groups was statistically similar. A non-significant reduction in the number of glial cells was observed in the CSD group compared to the cage-control one. The number of glial cells in the grid-floor group that received curcumin increased in comparison to cage-control animals (*P* = 0.0006), grid-floor control group (*P* = 0.0003), and CSD+curcumin (*P* = 0.01). The range of CE of the estimated glial cell number was 4–6% for the six groups (Fig. 3C).

### 3.5 The percentage of apoptotic cells

**Fig. 4:**
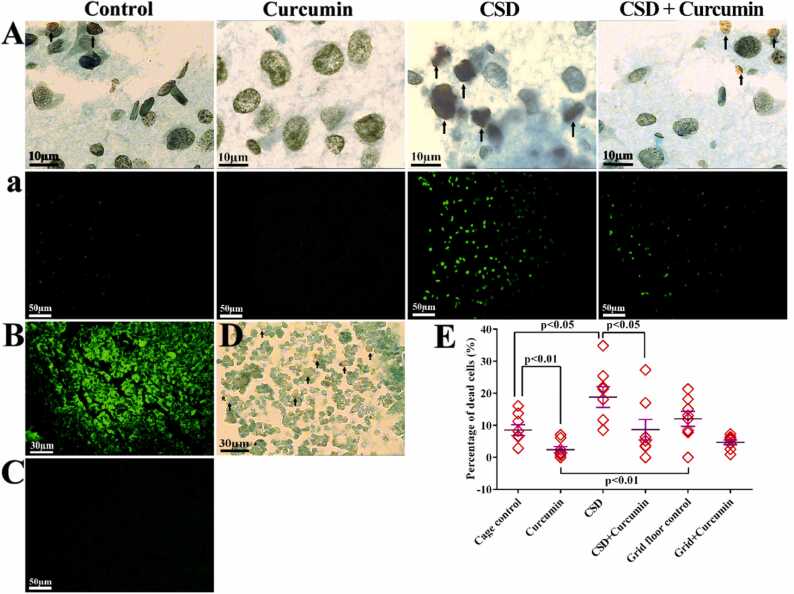
TUNEL assay. A: TUNEL-positive cells by light microscope and a: fluorescence microscope. Arrows indicate the apoptotic cells. Normal appearance and the low apoptotic cell can be seen in the control nucleus. In the curcumin-treated animals, the apoptotic cell was not observed. An obvious increase in the number of apoptotic cells was observed within the CSD animals compared with the control groups. The number of apoptotic cells in the CSD animals that received curcumin appeared to be similar to the control group. B&D: Positive control, the rat thymus with hydrocortisone-induced apoptosis thymocyte was used as the positive control. A large number of apoptotic cells can be seen in this tissue. C: The negative control, TdT enzyme was removed from the TdT reaction buffer that did not display TUNEL-positive cells. E: The mean ± SEM of apoptotic cell percentage in the different groups.

The percentage of TUNEL-positive neurons was similar in the grid-floor vs. cage-control groups and curcumin vs. grid-floor + curcumin ones (Fig. 4). The percentage of the TUNEL-positive neurons in the CSD group was significantly higher than the cage-control one (45.2 ± 1.19%, *P* = 0.04). Cell death in the curcumin-receiving groups, including CSD+curcumin (53.62 ± 0.24%, *P* < 0.05), curcumin (81.58 ± 1.87%, *P=*0.0005), and grid-floor+curcumin (69.64 ± 2.15%, *P* = 0.004) groups significantly decreased when compared to the CSD group, which may indicate protective effects of curcumin against apoptosis during CSD as well as in daily life.

### 3.6 The spatial arrangement of the parvocellular neurons

**Fig. 5:**
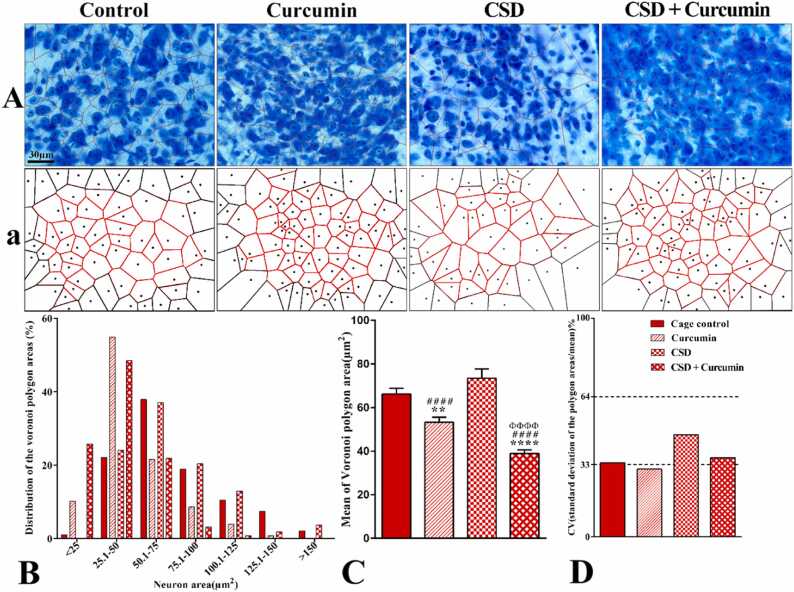
Representative photograph (A) and schematic (a) Voronoi tessellation of the PVH parvocellular neurons in different groups. (B) The distribution of the Voronoi polygon areas. (C) The mean of polygon areas for each neuron (µm^2^) in different groups. (D) The CV (coefficient of variation) of the spatial distribution of neurons in different groups: CV of 33–64% represented a random distribution (CSD and control group), and less than 33% had a regular pattern (curcumin-treated groups). ****P < 0.0001 and **P < 0.01 vs. Control; ^####^*P* < 0.0001 vs. the sleep-deprived; ^Φ Φ Φ Φ^*P* < 0.0001 vs. the curcumin group.

Based on the polygon area distribution, about 61% of the polygon areas in the control and CSD groups were placed in the range of 1–75 µm^2^. In fact, in both groups, the distribution of the polygon area was shifted to the right side of the graph (Fig. 5B). Almost similar range of polygon area distribution was detected in both healthy and CSD animals that received curcumin (86% and 96%, respectively). In these groups, the distribution of the polygon area was shifted to the left. The mean value of the polygon area of the neurons significantly increased in the CSD group compared to the curcumin and CSD+curcumin groups. Treatment with curcumin led to a significant decrease in this parameter in both healthy (*P* = 0.001) and CSD (*P* < 0.0001) animals compared to the control group (*P* < 0.0001, Fig. 5C). The mean value of the polygon area in the CSD+curcumin group was significantly smaller than the curcumin group (*P* < 0.0001, Fig. 5C).

The CV of the polygon areas was 33.85%, 30.91%, 46.65%, and 36.13% in the control, curcumin, CSD, and CSD+curcumin groups, respectively. These data indicated that the neuronal distribution in the curcumin group was regular (CV<33%) and it was very close to regular in the control group (33.85%), whereas the CSD groups had a random neuronal distribution (CV>33%, Fig. 5D). Feeding the CSD animals with curcumin shifted the distribution pattern toward regular. CV is classified based on the Brown-Forsythe test that indicates non-equal variances for the polygon areas. CV of the parvocellular neurons significantly differs in all groups (*F*_3, 575_ = 33.47, *P* < 0.0001).

### 3.7 The spatial arrangement of the magnocellular neurons

**Fig. 6:**
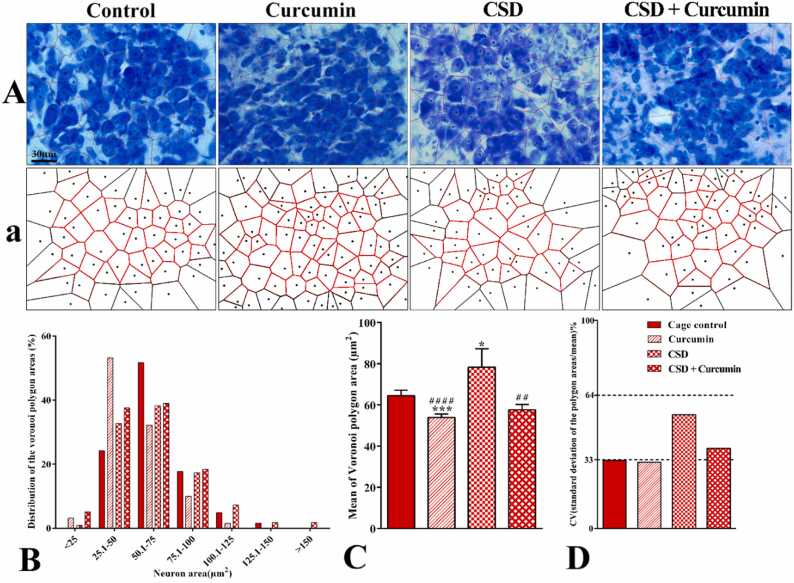
Representative photograph (A) and schematic. (a) Voronoi tessellation of the PVH magnocellular neurons in different groups. (B) The distribution of the Voronoi polygon areas. (C) The comparison of the mean of polygon areas for each neuron (µm^2^) in different groups. (D) The comparison of the CV of the spatial distribution of the neurons in different groups: CV (coefficient of variation) of 33–64% are with a random distribution (CSD and CSD+curcumin group), and less than 33% has a regular pattern (control and curcumin groups).*P < 0.05 and ***P < 0.001 vs. the control; ^##^*P* < 0.01 and^####^*P* < 0.0001 vs. the CSD group.

Based on the polygon area distribution, about 75.8% of the polygon areas in the control group and 71% in the CSD group are placed in the range of 1–75 µm^2^ (Fig. 6B). Both healthy and CSD animals that received curcumin also showed indistinguishable difference in the range of polygon area distribution (88.49% and 81.62%, respectively). In these groups, the distribution of the polygon area was shifted to the left. The mean coefficient of variation (CV) of the polygon areas was 32.68%, 31.83%, 54.7%, and 40.16% in the control, curcumin, CSD, and CSD + curcumin groups, respectively. These data showed that the distribution of the neurons in the control and curcumin-treated groups was regular (CV<33%), whereas the CSD group had a random neuronal distribution (CV>33%, Fig. 6C). Curcumin treatment of CSD animals improved the distribution pattern of the magnocellular neurons. CV of all groups revealed a significant difference in the magnocellular PVH neurons (*F*_3, 640_ = 13.9, *P* < 0.0001).

The mean value of the polygon area of the neurons significantly increased in the CSD group compared to the control group (*P* = 0.04, Fig. 6D). However, treatment with curcumin significantly decreased this parameter compared to the control group (*P* = 0.0006, Fig. 6D). Curcumin consumption decreased the neuronal areas in both healthy and CSD animals compared to the CSD animals (*P* < 0.001, Fig. 6D).

## 4 Discussion

The main findings of the current study revealed that CSD led to a decrease in PVH volume and the number of neurons and glial cells and an increase in the number of apoptotic neurons. For the first time, we also showed that the spatial pattern of PVH neurons in CSD animals changed to random. Besides, curcumin administration prevented the PVH damages induced by CSD.

Previous studies revealed that CSD reduced the tissue volume and led to a decrease in the cell number of different parts of the brain, including the dorsal respiratory nuclei in the brain stem (Kamali et al., 2017), hippocampus (Noorafshan et al., 2017), locus coeruleus (Jameie et al., 2019), medial prefrontal cortex (Noorafshan et al., 2017), and superior sympathetic ganglion (Erfanizadeh et al., 2020). In this study, tissue volume and cell number were changed in PVH.

Our result also confirmed a decrease in the number of neurons and glial cells in PVH in CSD animals, which eventually led to a reduction in PVH volume. This could be due to either an increase in neuronal death (Cao et al., 2019), or a decrease or cease in neurogenesis (Murata et al., 2018). Although we did not detect neurogenesis, an increase in neuronal apoptosis can be one of the reasons that account for a decrease in the tissue volume and cell count. However, we detected neuronal apoptosis elevation in CSD animals. There are controversial reports about the effect of sleep deprivation on the induction of cell death and degeneration. It depends on the type and duration of sleep deprivation. For example, Hipolide et al. have reported that sleep deprivation does not affect indices of necrosis or apoptosis in rat brains (Hipolide et al., 2002). Their animals have been deprived of sleep just for 96 h whereas our method is REM sleep deprivation for 21 days. Furthermore, Somarajan et al. in a study showed that REM sleep deprivation triggers the apoptosis intrinsic pathway in locus coeruleus (Somarajan et al., 2016). Jameie et al. have also reported that REM sleep deprivation decreased both neuron number and volume of the locus coeruleus. Biswas et al. have shown cell death in the medial preoptic area following REM sleep deprivation which is in line with our findings.

CSD has been shown to affect neuronal cytomorphology and structural proteins leading to neuronal apoptosis, which may be mediated by a boost in the level of the brain noradrenaline (Ranjan et al., 2010) by activating the mitochondrial intrinsic apoptotic pathway (Li et al. 2017). CSD has a general effect on all nerve cells in the brain and regulates most physiological processes in the body. Fifel et al. (2018) showed that neuronal apoptosis in PVH after CSD could be one of the reasons for abnormal behavior and mood disturbances (Fifel et al., 2018). Thus, it was necessary to investigate the effect of CSD on the rate of neuronal apoptosis in PVH, due to its special role in controlling stress, metabolism, growth, reproduction, immune, and autonomic functions (Qin et al., 2018).

CSD led to an increase in the spatial distance of both magno and parvocellular neurons and their random arrangement that may be attributed to cell death and a decrease in the number of neurons. Our TUNEL results also confirmed that cell death happened after CSD administration. Although curcumin did not have any positive or negative influence on neuronal distribution, it showed preventive impact when the animals with CSD experience were fed with it.

The administration of curcumin significantly prevented neuronal and glial cell loss, rate of neuronal death, and change in neuronal architecture in PVH during CSD. Oral administration of curcumin has been previously reported to suppress apoptosis, induce microglial activation, and decrease oxidative stress and histopathological damage in the brain (Abrahams et al., 2019). Our previous studies in the hippocampus (Noorafshan et al., 2018, 2017), medial Prefrontal Cortex (Kamali et al., 2017), and superior sympathetic nucleus (Erfanizadeh et al., 2020) have revealed the neuroprotective impact of curcumin. As a neuroprotectant, curcumin exerts its effects through some mechanisms such as modulating transcription factors, inflammatory cytokines, kinases, growth factors, and antioxidants. Furthermore, curcumin attenuated the toxicity of neurons through the inhibition of ROS, mitochondrial protection, and anti-apoptotic mechanisms. Curcumin suppresses DNA fragmentation and apoptosis through elevating the expression of the phosphorylated extracellular signal-regulated kinases (ERK), releasing Bcl-2 (which has an anti-apoptosis effect) and inhibiting caspase 3 (inducer DNA fragmentation) (Li et al., 2017). Moreover, the progression of neurodegenerative diseases can be attenuated by the capacity of curcumin to decrease neuro-inflammation (Chin, 2016).

## 5 Conclusion

CSD, for whatever reason, has serious effects on PVH volume and mortality of the neuronal and glial cells. We showed that curcumin protected the normal PVH structure and spatial pattern of magno and parvocellular neurons in CSD rats.

## Ethical statement

All animal procedures were approved by the Institutional Animal Care and Use Committee at the School of Shiraz University of Medical Sciences, Shiraz, Iran (IR.SUMS.REC.1396. S630), and conducted in accordance with the National Institutes of Health’s Guide for Care and Use of Laboratory Animals and the Animal Research: Reporting in Vivo Experiments (ARRIVE) Guidelines.

## CRediT authorship contribution statement

**A.N. and M.R.N.** designed and supervised the study. **M.E.** performed histological evaluations, wrote the paper draft, and prepared the figures and graphs. **T.T., S.K., and M.R.N.** analyzed the data and edited the paper. All authors approved the final version of the article.

## Declaration of Competing Interest

The authors declare that they have no competing financial interests.

## References

[bib1] Abrahams S., Haylett W.L., Johnson G., Carr J.A., Bardien S. (2019). Antioxidant effects of curcumin in models of neurodegeneration, ageing, oxidative and nitrosative stress: a review. Neuroscience.

[bib2] Aguirre C.C. (2016). Sleep deprivation: a mind-body approach. Curr. Opin. Pulm. Med..

[bib3] Amaral F.G. d, Cipolla-Neto J. (2018). A brief review about melatonin, a pineal hormone. Arch. Endocrinol. Metab..

[bib4] Amrein I., Slomianka L., Lipp H.P. (2004). Granule cell number, cell death and cell proliferation in the dentate gyrus of wild‐living rodents. Eur. J. Neurosci..

[bib5] Biswas, S., Mishra, P., & Mallick, B.J.N. (2006). Increased apoptosis in rat brain after rapid eye movement sleep loss. *142*(2), 315–331. 10.1016/j.neuroscience.2006.06.026 16887278

[bib6] Bjugn, R., & Gundersen, H.J.G.J.J. o C.N. (1993). Estimate of the total number of neurons and glial and endothelial cells in the rat spinal cord by means of the optical disector. *328*(3), 406–414. 10.1002/cne.903280307 8440788

[bib7] Bock M., Tyagi A.K., Kreft J.-U., Alt W. (2010). Generalized voronoi tessellation as a model of two-dimensional cell tissue dynamics. Bull. Math. Biol..

[bib8] Cao Y., Li Q., Liu L., Wu H., Huang F., Wang C., Zhou Q. (2019). Modafinil protects hippocampal neurons by suppressing excessive autophagy and apoptosis in mice with sleep deprivation. Br. J. Pharmacol..

[bib9] Chin K.-Y. (2016). The spice for joint inflammation: anti-inflammatory role of curcumin in treating osteoarthritis. Drug Des. Dev. Ther..

[bib10] da Silva Rocha-Lopes, J., Machado, R.B., & Suchecki, D.J.M.N. (2018). Chronic REM sleep restriction in juvenile male rats induces anxiety-like behavior and alters monoamine systems in the amygdala and hippocampus. *55*, 2884–2896. 10.1007/s12035-017-0541-3 28455701

[bib11] Deniz Ö.G., Altun G., Kaplan A.A., Yurt K.K., von Bartheld C.S., Kaplan S. (2018). A concise review of optical, physical and isotropic fractionator techniques in neuroscience studies, including recent developments. J. Neurosci. Methods.

[bib12] During E., Kawai M. (2017). Sleep and Neurologic Disease.

[bib13] Duyckaerts C., Godefroy G. (2000). Voronoi tessellation to study the numerical density and the spatial distribution of neurones. J. Chem. Neuroanat..

[bib14] Erfanizadeh M., Noorafshan A., Namavar M.R., Karbalay-Doust S., Talaei-Khozani T. (2020). Curcumin prevents neuronal loss and structural changes in the superior cervical (sympathetic) ganglion induced by chronic sleep deprivation, in the rat model. Biol. Res..

[bib15] Fan K., Yang J., Gong W.-Y., Pan Y.-C., Zheng P., Yue X.-F. (2021). NLRP3 inflammasome activation mediates sleep deprivation-induced pyroptosis in mice. PeerJ.

[bib16] Fifel K., Meijer J.H., Deboer T. (2018). Long-term effects of sleep deprivation on neuronal activity in four hypothalamic areas. Neurobiol. Dis..

[bib17] Füzesi T., Daviu N., Cusulin J.I.W., Bonin R.P., Bains J.S. (2016). Hypothalamic CRH neurons orchestrate complex behaviours after stress. Nat. Commun..

[bib18] García-Cabezas, M.Á., John, Y.J., Barbas, H., & Zikopoulos, B.J.F. i n (2016). Distinction of neurons, glia and endothelial cells in the cerebral cortex: an algorithm based on cytological features. *10*, 107. 10.3389/fnana.2016.00107 PMC5088408 27847469

[bib19] Hipolide, D.C., D'ALMEIDA, V., Raymond, R., Tufik, S., & Nobrega, J.N.J.I. j o n (2002). Sleep deprivation does not affect indices of necrosis or apoptosis in rat brain. *112*(2), 155–166. 10.1080/00207450212022 12325404

[bib20] Howard V., Reed M. (2004). Unbiased Stereology: Three-dimensional Measurement in Microscopy.

[bib21] Jameie S.B., Mesgar S., Aliaghaei A., Raoofi A., Amini M., Khodagholi F., Sadeghi Y. (2019). Neuroprotective effect of exogenous melatonin on the noradrenergic neurons of adult male rats’ locus coeruleus nucleus following REM sleep deprivation. J. Chem. Neuroanat..

[bib22] Kamali A.-M., Noorafshan A., Karimi F., Karbalay-Doust S., Nami M. (2017). The impact of chronic sleep restriction on neuronal number and volumetric correlates of the dorsal respiratory nuclei in a rat model. Sleep.

[bib23] Kristiansen S.L.B., Nyengaard J.R. (2012). Digital stereology in neuropathology. Apmis.

[bib24] Leng Y., Musiek E.S., Hu K., Cappuccio F.P., Yaffe K. (2019). Association between circadian rhythms and neurodegenerative diseases. Lancet Neurol..

[bib25] Li C.Y., Zhang L., Li J., Qi C.L., Li D.Y., Liu X., Qu X. (2017). Effect of endogenous arginine-vasopressin arising from the paraventricular nucleus on learning and memory functions in vascular dementia model rats. BioMed Res. Int..

[bib26] Li X., Feng K., Li J., Yu D., Fan Q., Tang T., Wang X. (2017). Curcumin inhibits apoptosis of chondrocytes through activation ERK1/2 signaling pathways induced autophagy. Nutrients.

[bib27] Lim J., Dinges D.F. (2010). A meta-analysis of the impact of short-term sleep deprivation on cognitive variables. Psychol. Bull..

[bib28] Liu Y., Li Y., Yang B., Yu M., Zhang X., Bi L., Xu H. (2020). Glutamatergic neurons of the paraventricular nucleus are critical for the control of wakefulness. Neuroscience.

[bib29] Lu J., Zhang Y.-H., Chou T.C., Gaus S.E., Elmquist J.K., Shiromani P., Saper C.B. (2001). Contrasting effects of ibotenate lesions of the paraventricular nucleus and subparaventricular zone on sleep–wake cycle and temperature regulation. J. Neurosci..

[bib30] Machado R.B., Tufik S., Suchecki D. (2013). Role of corticosterone on sleep homeostasis induced by REM sleep deprivation in rats. PLoS One.

[bib31] Machluf Y., Gutnick A., Levkowitz G. (2011). Development of the zebrafish hypothalamus. Ann. N. Y. Acad. Sci..

[bib32] Miklós I.H., Kovács K.J. (2012). Reorganization of synaptic inputs to the hypothalamic paraventricular nucleus during chronic psychogenic stress in rats. Biol. Psychiatry.

[bib33] Moreno-Villanueva, M., Scheven, G.V., Feiveson, A., Bürkle, A., Wu, H., & Goel, N. (2018). Original Article The degree of radiation-induced DNA strand breaks is altered by acute sleep deprivation and psychological stress and is associated with cognitive performance in humans. 10.1093/sleep/zsy067 29596659

[bib34] Moroni, R.F., Inverardi, F., Regondi, M.C., Panzica, F., Spreafico, R., & Frassoni, C.J.E. (2008). Altered spatial distribution of PV‐cortical cells and dysmorphic neurons in the somatosensory cortex of BCNU‐treated rat model of cortical dysplasia. *49*(5), 872–887. 10.1111/j.1528-1167.2007.01440.x 18076647

[bib35] Murata Y., Oka A., Iseki A., Mori M., Ohe K., Mine K., Enjoji M. (2018). Prolonged sleep deprivation decreases cell proliferation and immature newborn neurons in both dorsal and ventral hippocampus of male rats. Neurosci. Res..

[bib36] Mutiah R., Griana T.P., Ula Q.N., Andhyarto Y. (2016). The effect of Calotropis gigantea leaves extract on fibrosarcoma growth and caspase 3 expression. Int. J. Pharm. Clin. Res..

[bib37] Namavar, M., Ghalavandi, M., & Bahmanpour, S.J.J. o C.N. (2020). The effect of glutathione and buserelin on the stereological parameters of the hypothalamus in the cyclophosphamide-treated mice. 110, 101871. 10.1016/j.jchemneu.2020.101871 33039509

[bib38] Noorafshan A., Karimi F., Kamali A.-M., Karbalay-Doust S., Nami M. (2018). Could curcumin protect the dendritic trees of the CA1 neurons from shortening and shedding induced by chronic sleep restriction in rats?. Life Sci..

[bib39] Noorafshan A., Karimi F., Karbalay-Doust S., Kamali A.M. (2017). Using curcumin to prevent structural and behavioral changes of medial prefrontal cortex induced by sleep deprivation in rats. EXCLI J..

[bib40] Odaka C., Mizuochi T. (2002). Macrophages are involved in DNA degradation of apoptotic cells in murine thymus after administration of hydrocortisone. Cell death Differ..

[bib41] Pakkenberg B., Gundersen H. (1988). Total number of neurons and glial cells in human brain nuclei estimated by the disector and the fractionator. J. Microsc..

[bib42] Paxinos G., Watson C. (2006). The Rat Brain in Stereotaxic Coordinates: Hard Cover Edition.

[bib43] Qin C., Li J., Tang K. (2018). The paraventricular nucleus of the hypothalamus: development, function, and human diseases. Endocrinology.

[bib44] Quey R., Renversade L. (2018). Optimal polyhedral description of 3D polycrystals: method and application to statistical and synchrotron X-ray diffraction data. Comput. Methods Appl. Mech. Eng..

[bib45] Ranjan A., Biswas S., Mallick B.N. (2010). Cytomorphometric changes in the dorsal raphe neurons after rapid eye movement sleep deprivation are mediated by noradrenalin in rats. Behav. Brain Funct..

[bib46] Somarajan B.I., Khanday M.A., Mallick B.N. (2016). Rapid eye movement sleep deprivation induces neuronal apoptosis by noradrenaline acting on Alpha1 adrenoceptor and by triggering mitochondrial intrinsic pathway. Front. Neurol..

[bib47] Somarajan, B.I., Khanday, M.A., & Mallick, B.N.J.F. i n (2016). Rapid eye movement sleep deprivation induces neuronal apoptosis by noradrenaline acting on Alpha1 adrenoceptor and by triggering mitochondrial intrinsic pathway. *7*, 25. 10.3389/fneur.2016.00025 PMC4779900 27014180

[bib48] Sotoudeh, N., Namavar, M., Zarifkar, A., & Heidarzadegan, A.J.I. r (2020). Age-dependent changes in the medial prefrontal cortex and medial amygdala structure, and elevated plus-maze performance in the healthy male Wistar rats. 9, 183–194. 10.1016/j.ibror.2020.08.002 PMC7452646 32885088

[bib49] Teclemariam-Mesbah R., Kalsbeek A., Buijs R.M., Pévet P. (1997). Oxytocin innervation of spinal preganglionic neurons projecting to the superior cervical ganglion in the rat. Cell Tissue Res..

[bib50] Uylings H., Malofeeva L., Bogolepova I., Jacobsen A., Amunts K., Zilles K. (2005). No postnatal doubling of number of neurons in human Broca’s areas (Brodmann areas 44 and 45)? A stereological study. Neuroscience.

[bib51] van Horssen, P., van den Wijngaard, J.P., Brandt, M., Hoefer, I.E., Spaan, J.A., & Siebes, M. (2013). Perfusion territories subtended by penetrating coronary arteries increase in size and decrease in number towards the subendocardium. *American Journal of Physiology-Heart and Circulatory Physiology*. 10.1152/ajpheart.00584.2013 24363303

[bib52] Verkhratsky A., Ho M.S., Zorec R., Parpura V. (2019). Neuroglia in Neurodegenerative Diseases.

[bib53] Zhao J., Yu S., Zheng W., Feng G., Luo G., Wang L., Zhao Y. (2010). Curcumin improves outcomes and attenuates focal cerebral ischemic injury via antiapoptotic mechanisms in rats. Neurochem. Res..

[bib54] Zhou H.-Y., Sun Y.-Y., Chang P., Huang H.-C. (2022). Curcumin inhibits cell damage and apoptosis caused by thapsigargin-induced endoplasmic reticulum stress involving the recovery of mitochondrial function mediated by mitofusin-2. Neurotox. Res..

[bib55] Zhu B., Shi C., Park C.G., Zhao X., Reutrakul S. (2019). Effects of sleep restriction on metabolism-related parameters in healthy adults: a comprehensive review and meta-analysis of randomized controlled trials. Sleep Med. Rev..

[bib56] Zhang F., Zhou Y., Chen H., Jiang H., Zhou F., Lv B., Xu M. (2022). Curcumin Alleviates DSS-Induced Anxiety-Like Behaviors via the Microbial-Brain-Gut Axis. Oxidative Med. Cell. Longev..

